# Genome-wide analysis of the cotton G-coupled receptor proteins (GPCR) and functional analysis of GTOM1, a novel cotton GPCR gene under drought and cold stress

**DOI:** 10.1186/s12864-019-5972-y

**Published:** 2019-08-14

**Authors:** Pu Lu, Richard Odongo Magwanga, Joy Nyangasi Kirungu, Qi Dong, Xiaoyan Cai, Zhongli Zhou, Xingxing Wang, Yanchao Xu, Yuqing Hou, Renhai Peng, Kunbo Wang, Fang Liu

**Affiliations:** 1 State Key Laboratory of Cotton Biology/Institute of Cotton Research, Chinese Academy of Agricultural Science (ICR-CAAS), Anyang, 455000 Henan China; 2 School of Physical and Biological Sciences (SPBS), Main campus, Jaramogi Oginga Odinga University of Science and Technology, P.O Box 210-40601, Bondo, Kenya; 3 Research Base in Anyang Institute of Technology, State Key Laboratory of Cotton Biology/Anyang Institute of technology, Anyang, 455000 Henan China; 4 School of Agricultural Sciences, Zhengzhou University, 450001 Henan China

**Keywords:** Cotton GPCR gene, Drought stress, Cold stress, GO analysis, miRNAs, Oxidant and antioxidant enzymes, Transgenic lines

## Abstract

**Background:**

The efficient detection and initiation of appropriate response to abiotic stresses are important to plants survival. The plant G-protein coupled receptors (GPCRs) are diverse membranous proteins that are responsible for signal transduction.

**Results:**

In this research work, we identified a novel gene of the GPCR domain, transformed and carried out the functional analysis in Arabidopsis under drought and cold stresses. The transgenic lines exposed to drought and cold stress conditions showed higher germination rate, increased root length and higher fresh biomass accumulation. Besides, the levels of antioxidant enzymes, glutathione (GSH) and ascorbate peroxidase (APX) exhibited continuously increasing trends, with approximately threefold higher than the control, implying that these ROS-scavenging enzymes were responsible for the detoxification of ROS induced by drought and cold stresses. Similarly, the transgenic lines exhibited stable cell membrane stability (CMS), reduced water loss rate in the detached leaves and significant values for the saturated leaves compared to the wild types. Highly stress-responsive miRNAs were found to be targeted by the novel gene and based on GO analysis; the protein encoded by the gene was responsible for maintaining an integral component of membrane. In cotton, the virus-induced gene silencing (VIGS) plants exhibited a higher susceptibility to drought and cold stresses compared to the wild types.

**Conclusion:**

The novel *GPCR* gene enhanced drought and cold stress tolerance in transgenic Arabidopsis plants by promoting root growth and induction of ROS scavenging enzymes. The outcome showed that the gene had a role in enhancing drought and cold stress tolerance, and can be further exploited in breeding for more stress-resilient and tolerant crops.

**Electronic supplementary material:**

The online version of this article (10.1186/s12864-019-5972-y) contains supplementary material, which is available to authorized users.

## Background

Plant bodies are complex and due to ever-changing environmental conditions, well-coordinated internal activities are vital for their survival [1]. Drought stress is one of the major abiotic factors which have caused massive loses in crops [2]. The global climate change has made the situation worse, being temperature stress has also become a major area of concern for the researchers worldwide [3]. Being environmental factors are dynamic and require complex response mechanisms by plants, the evolution of various transcription factors has revolutionist plants adaption and acclimation to various forms of abiotic stress factors [4]. Life forms emerged from simple unicellular organisms, and due to their body sizes, no much coordination was required, but the emergence of the complex eukaryotic organisms, they are highly dependent on the ability of their cells not only to communicate with each other but also to their external environment [5]. In the past 15 years, researchers have made numerous discoveries on plant signal transduction towards their environments. The membrane-bound receptors responsible for the detection of endocellular messenger molecules and exocellular messages are majorly grouped into four or five protein families, and the most common one is the G-protein coupled receptor (GPCR) family [6].

The GPCR proteins form the largest and most diverse family of the membrane proteins in eukaryotes, with a unique feature of seven transmembrane (7-TM) receptors, also referred to as heptahelical [7]. GPCR protein families are responsible for the transduction of signals across the cell membrane, which controls varied physiological and biological processes, thus are highly favored as drug targets more so in animals. In higher animals, the estimated number of GPCR protein families believed to be harbored in their genome ranges between 1,000 to 2,000 [8]. The GPCR proteins are known to be among the earlier forms of proteins responsible for signal transduction, in plants [9], yeast [10] and slime mold [11], as well as in protozoa and the earliest diploblastic metazoa organisms [12]. The structural similarities among the members of the GPCR proteins are debatable, being sequences comparison between different members of the GPCR proteins has revealed the existence of different receptors with no sequence similarities among the various GPCR proteins, though the common feature is the presence of 7-TM helices [13]. In addition to the differences in their sequences, the GPCR proteins also differ in their protein length and function of their intracellular and extracellular domains which are the C-terminal and N-terminal, respectively, in addition to the functions of their intracellular loops [14].

Recent studies have shown that the GPCR proteins function as a signal transducer with an immense role in enhancing abiotic stress tolerance [15]. In our previous studies on the *GPCR* genes, we found that the cotton *Gh_A07G0740 (GhTOM1)* overexpressed Arabidopsis plants exhibited a higher tolerance to salt stress compared to the wild types [16]. Moreover, in *Arabidopsis thaliana*, two members of the GPCR family, such as GTG_1 and GTG_2, are novel types of GPC-GTPases, functions as abscisic acid (ABA) receptors [17]. ABA is a plant phytohormone with diverse roles in the regulation of plant response to stresses, in addition to its growth and development [18]. In addition, several proteins have been found to function as ABA receptors, for example, G-protein coupled receptor 2 (GPCR2), magnesium-chelatase H subunit (CHLH) and flowering time control protein A (FCA) [19].

Typical GPCR proteins have 7-TM domain receptors that are induced by ligands and interact with G subunits, upon signal detection, GPCRs undergoes a transformation, to allow the G-alpha subunit to exchange guanosine diphosphate (GDP) for guanosine triphosphate (GTP). Once activated, the heterotrimeric complex breaks into GTP-G alpha and G beta-gamma dimer, in which the two none related elements can interact with downstream signaling effectors. Despite the immense role of GPCR proteins in living organisms, little information is available about their distribution, abundance, and the roles they play in plants under abiotic stress conditions. In the model plant, *A. thaliana*, a smaller number of GPCR proteins have been investigated, such as 1 G alpha (GPA1), a single G-beta (AGB1), 3 G gamma subunits such as AGG designated as AGG1 to AGG3, and a single regulator of the G-protein signalling [20]. A number studies have associated the GPCR proteins to have a role in the enhancing germination of the seeds and their growth, stomatal regulation, plant defense responses, and development of various plant organs such as the roots, rosette leaf, flower, and silique [21]. In addition, GPCR proteins functions have been elucidated in rice, rice plant being semi-hydrophytic type of plant, its growth and production is highly dependent on the availability of water, the induction of rice GPCR proteins under salt, ABA, drought and cold stresses showed that the GPCR proteins have a regulatory role under abiotic stress [15].

The GPCR proteins are diverse, and several subfamilies have been proposed to belong to the 15 mildew-resistance locus O (MLO) proteins, G-protein-coupled receptor 1 (GCR1), tobamovirus replication protein (TOM), five heptahelical protein (HHP) proteins [22], *A. thaliana* regulator of G-protein signaling 1 (AtRGS1) and candidate GPCR (CAND) proteins [23]. In all the various subfamilies of the plant GPCR proteins, no common agreement has been reached among researchers, whether they are plant’s GPCR proteins or not, being the plants GPCR proteins evolved at a rapid pace [24]. Furthermore, despite the debate whether GCR1 is a functional domain or not, *AtGCR1* is known to be the candidate plants GPCR protein and has been found to play a role in enhancing stress tolerance, hormones biosynthesis, plant secondary metabolite synthesis and phosphate starvation tolerance in plants, moreover, knockdown of *AtGCR1* in Arabidopsis significantly reduced their ability to tolerate effects of various abiotic stress factors [25]. In all the subfamilies, a lot has been done, but little is known about the *TOM* genes, despite being one of the plant GPCR proteins. The proteins encoding the *TOM1* genes have been proved to be members of GPCRs, and have been found to interact with the Gα subunit (GPA1) [26].

Cotton being an important crop for the production of natural fiber and edible oil, its production is on the steady decline due to various abiotic stress factors, compounded by ever reducing arable land suitable for crop production. The increased pressure on arable land due to human demand for settlement as a result of increased population, non-edible crops are being pushed to the drier regions and highly saline soils. Therefore, understanding of how plants detect and respond to environmental stress factors is critical. We undertook to investigate the functional roles of the cotton GPCR proteins under drought and cold stress conditions, in which a novel cotton *GPCR* gene (*Gh_A07G0747 (GhTOM1)* was transformed into the model plant, *A. thaliana*, and carried out analysis under drought and cold stress conditions. In addition, the gene was silenced, and then reintroduced it to upland cotton seedling to understand their possible role in cotton under drought and cold stress conditions.

## Results

### Identification, phylogenetic analysis and physiochemical properties of the proteins encoded by the *TOM* genes in cotton

A total of 36 proteins encoded by the *TOM* genes were identified in cotton, with 16, 11 and 9 proteins in *G. hirsutum, G. raimondii* and *G. arboreum*. The numbers of TOM proteins were relatively low compared to other plant proteins such as cyclin dependent kinase (CDK) [27], late embryogenesis abundant (LEA) proteins (LEA) [28] and or the multidrug and toxic compound extrusion [29]. The low numbers could perhaps mean that the genes have lost their function throughout plants evolution or they are new sets of genes that are being evolved by plants as a result of drastic changes in the environmental conditions. The various properties of the proteins encoded by the *TOM* genes in the three cotton species exhibited variations, in which the highest isoelectric point (*pI*) values was 10.33 and the lowest *pI* value was 6.501; similarly, the GRAVY values ranged from 0.337 to a maximum value of 0.753, none of the proteins had GRAVY values below zero an indication that the proteins encoded by the *TOM* genes of the GPCR domain are all hydrophilic. Moreover, the molecular weights 10.049 kDa to 44.238 kDa (Additional file 1: Table S1). Hydrophilic proteins have been found to have diverse roles in plants, and are critical in enhancing stress tolerance. For instance, RD29A and RD29B encode the very hydrophilic proteins and are critical in enhancing stress tolerance in plants [30]. The entire cotton TOM proteins were classified into four groups (Additional file 3: Figure S1), the result was similar to our previous classification in which only TOM proteins were used, and due to the low numbers, only three groups were formed [16]. Finally, in the determination of the subcellular localization prediction for the proteins encoded by the *GPCR* genes, all were found to be embedded at the plasma membrane, results which were in agreement to our previous prediction and experimental determination of the subcellular localization of the GPCR proteins in the cell [16].

### miRNA analysis, *Cis-*regulatory element, and gene ontology (GO) prediction

*Cis-*regulatory element analysis, miRNA prediction and gene ontology (GO) prediction, revealed that the cotton GPCR of the TOM1 subfamily has a role in enhancing abiotic stress tolerance. In the miRNA prediction, 5 miRNAs were found to target 7 genes. Only one gene was found to be targeted by more than one miRNA, *Gh_A13G0596* was targeted by two miRNAs; ghr-miR156c and ghr-miR7488, the rest of the genes were targeted by one miRNA. Uniquely, three miRNAs targeted more than one gene, for instance, ghr-miR156c targeted *Gh_A13G0596* and *Gh_D13G0530*, ghr-miR172 targeted *Gh_D05G1613* and *Gh_A05G1440*, and ghr-miR7510b targeted *Gh_D07G0809* and *Gh_A07G0747*. In all the five (5) miRNAs, ghr-miR7488 and ghr-miR7508 specifically targeted *Gh_A13G0596* and *Gh_A10G0365*, respectively. The various miRNAs detected in this study have been previously found to have a functional role in various abiotic stress factors in plants, for instance ghr-miR156c has been previously found to be highly upregulated under salt and drought stress conditions in plants [28].

In the analysis of the *Cis-*regulatory element on the promoter regions of the coding sequence of the cotton TOM of the GCPR domain, several functions geared towards enhancing abiotic and biotic tolerances in plants were detected. In the coding region for the novel gene, *Gh_A07G0747* (GhTOM1), the following *Cis-*regulatory elements were detected: ABRELATERD1 (ACGTG), inducted by dehydration stress and dark-induced senescence; ACGTATERD1 (ACGT), early responsive to dehydration; CBFHV (RYCGAC), responsible for cold acclimation; DPBFCOREDCDC3 (ACACNNG), can be induced by ABA/ABA-responsive and embryo-specification elements among others. Among all the *Cis-*regulatory elements detected for the novel gene, *Gh_A07G0747*, MYCCONSENSUSAT (CANNTG), with the primary role of MYB recognition site transcriptional activators in ABA signaling (Additional file 2: Table S2 and Additional file 4: Figure S2A). The detection of these *Cis-*regulatory elements showed that the gene has a functional role in enhancing stress tolerance in cotton plants.

In the analysis of the entire upland cotton *TOM* genes of the GCPR domain, only two GO functions were detected, GO: 0016021(integral component of membrane) and GO: 0051301 (cell division), and their two functions were also detected for cellular component (CC) and biological process (BP), respectively (Additional file 4: Figure S2B). In early analysis of the various genes found to be conserved between a more drought tolerant *G. tomentosum* and highly drought susceptible upland cotton genotype (*G. hirsutum)*, and in their backcross population (BC_2_F_2_), a number of GO functions were detected among which were those with integral role in the membrane and membrane part [31].

### RNA sequence and real time quantitative polymerase chain reaction (RT-qPCR) validation of the overexpressed gene under drought and cold stress conditions

The analysis of the RNA sequence data obtained from our institute showed that the entire upland cotton, *TOM* genes of the GPCR domains were clustered into three groups, in relation to their expression levels under drought and cold stress conditions. In group one, 5 genes were significantly upregulated across the various tissues. Members of group 2 were either downregulated or not expressed at except one gene, *Gh_D04G1878* which was partially upregulated at 12 h post exposure to drought stress condition. Lastly, the members of group 3 exhibited differential expression, though one gene, *Gh_Sca207201G01* showed insignificant expression across the tissues (Additional file 5: Figure S3A-B). The novel gene, *Gh_A07G0747* was highly upregulated in all the tissues tested under drought and cold stresses, and in verifying its expression through RT-qPCR, the gene showed abundance in both vegetative and reproductive tissues of the upland cotton, though was higher in stamen than the rest of the tissues (Additional file 5: Figure S3C). In the transformation of the model plant, *A. thaliana*, 10 lines were successfully transformed (Additional file 5: Figure S3D). And out of them, line 3, line 7 and line 9 showed the highest overexpression levels of the novel gene (Additional file 5: Figure S3E).

### Evaluation of physiological traits under drought and cold stress conditions

**Fig. 1:**
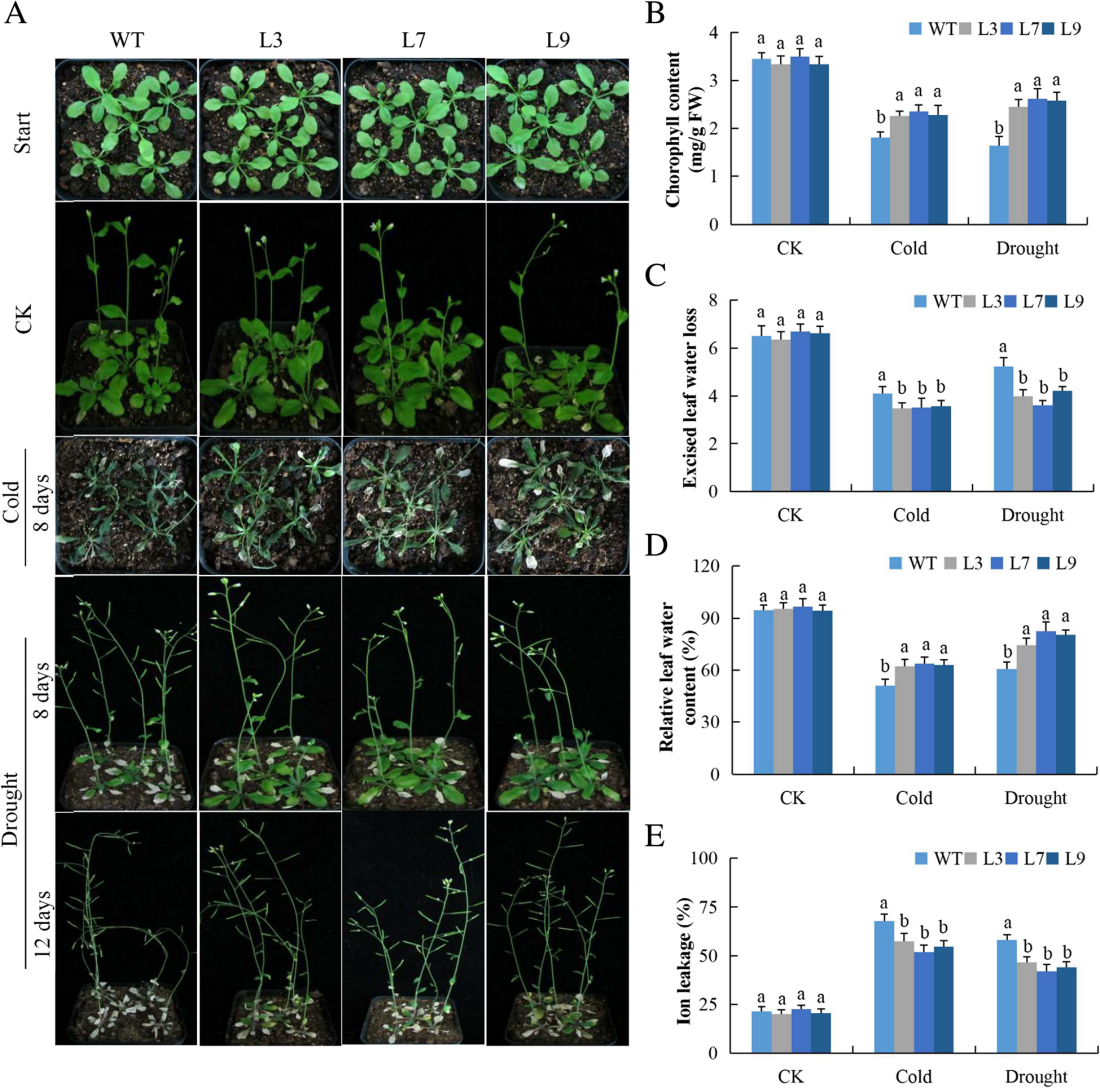
Physiological traits evaluation in *Gh_A07G0747 (GhTOM1)* overexpressed lines under drought and cold stress conditions: **a** *Gh_A07G0747 (GhTOM1)* overexpressed lines and wild type under stress conditions. **b** Quantitative determination of chlorophyll content. **c** Determination of excised leaf water loss (ELWL). **d** Quantitative determination of relative leaf water content (RLWC). **e** Quantitative determination of cell membrane stability (CMS) as ion leakage concentration in leaves of wild-type and both overexpressed after 8-day post stress exposure. In (**b**, **c**, **d** and **e**), each experiment was repeated three times. Bar indicates standard error (SE). Different letters indicate significant differences between wild-type and OE lines (ANOVA; *p* < 0.05). CK: normal conditions

The overexpressed lines and the wild types were treated with drought and cold stress conditions and their physiological parameters were evaluated. Chlorophyll content was significantly higher in the overexpressed lines, and the wild type leaves appeared chlorotic (Fig. 1a-b). The level of chlorophyll content was much higher in the Gh*_A07G0747 (TOM1)* gene overexpressed lines under drought stress as compared to cold stress. There was a sharp decline in chlorophyll content in the leaves of the wild type both in drought and cold stresses compared to normal conditions, showing that wild type plants featured a higher level of oxidative stress compared to the transgenic lines. We further evaluated excised leaf water loss (ELWL), cell membrane stability (CMS), and saturated leaf weight (SLW) in the leaf tissues of *Gh_A07G0747 (TOM1)* gene overexpressed lines and wild lines under drought and cold stress conditions. The values for the SLW were significantly higher among the *Gh_A07G0747 (TOM1)* gene overexpressed lines but significantly lower in the leaves of the wild types under drought and cold stress conditions (Fig. 1c). Similarly, the excised leaf water loss rate was significantly higher in the leaves of the wild type lines while significantly lower among the leaves obtained from the transgenic lines (Fig. 1d). The results showed that the *Gh_A07G0747 (TOM1)* gene overexpressed lines had a significantly high level of CMS as determined through ion leakage, the lower the level of ion leakage the stable the cell membrane and vice versa (Fig. 1f). The high CMS and SLW among the *Gh_A07G0747 (TOM1)* gene overexpressed lines showed that the gene had a potential role in increasing the capacity to tolerate the effects caused due to exposure to drought and cold stress conditions. In addition, the low rate of water loss as exhibited by the excised leaf from the *Gh_A07G0747 (TOM1)* gene overexpressed lines further indicates that the plant had a higher ability to regulate the stomatal conductance, thereby minimizing the rate of water loss through evapotranspiration. Drought and cold stress factors reduce plant growth and development by changing the chemical and physical composition of cell membranes, cause electrolyte leakage, decrease protoplasmic streaming and change in cell metabolism [32].

### Evaluation oxidant and antioxidant enzymes in *Gh_A07G0747 (TOM1*) gene overexpressed lines and wild types under drought and cold stress conditions

**Fig. 2:**
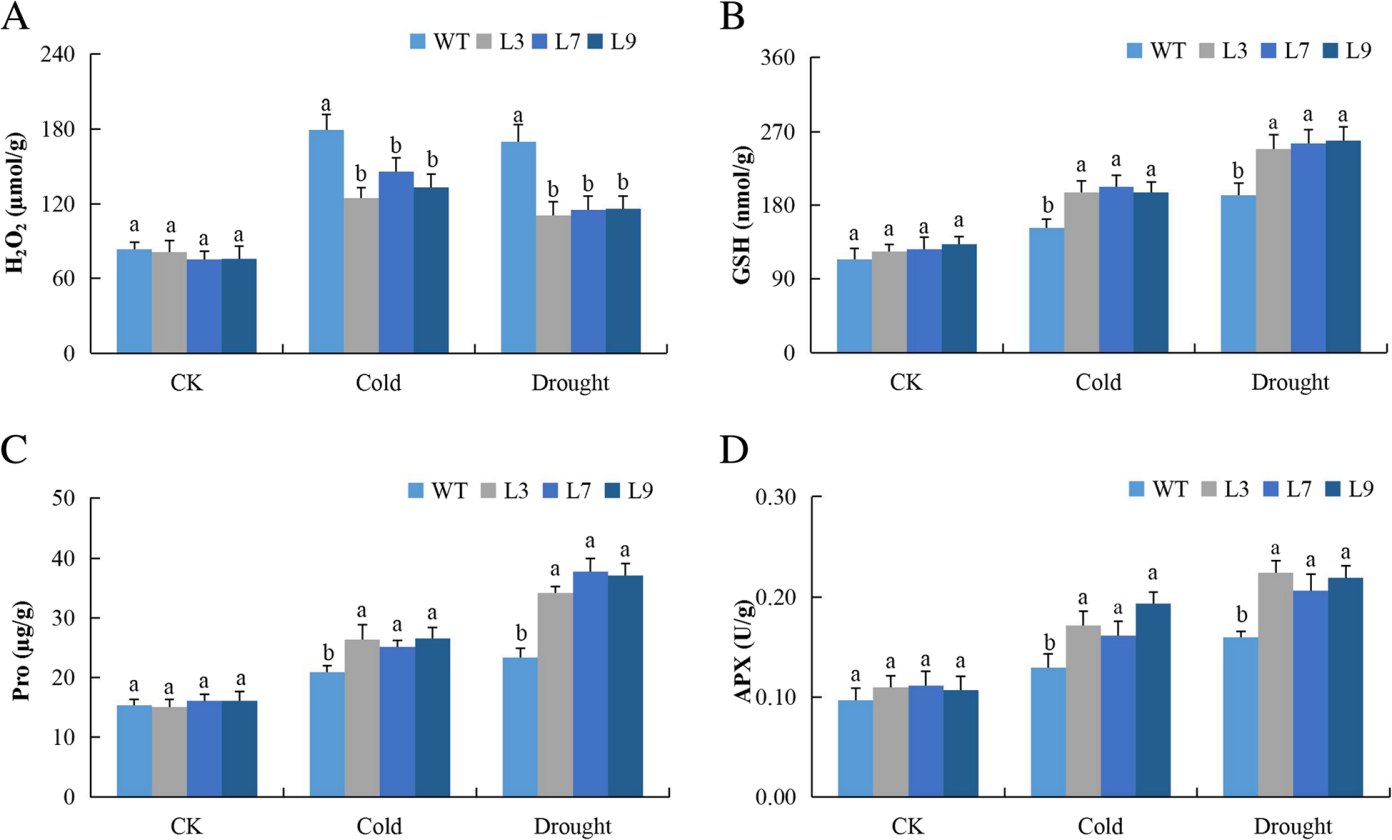
Determination of oxidants and antioxidants concentration levels in *Gh_A07G0747 (GhTOM1)* transgenic lines under drought and cold stress conditions: **a** Quantitative determination of hydrogen peroxide (H_2_O_2_) concentration. **b** Quantitative determination of glutathione (GSH) concentration. **c** Quantitative determination of proline content. **d** Quantitative determination of ascorbate peroxidase (APX) in leaves of wild-type and both overexpressed lines after 8-day post stress exposure. In (**a**, **b**, **c** and **d**), each experiment was repeated three times. Bar indicates standard error (SE). Different letters indicate significant differences between wild-type and OE lines (ANOVA; *p* < 0.05). CK: normal conditions

The concentrations of oxidant and antioxidant enzymes in the leave of the plants under stress conditions shows the ability to tolerate the effects caused by the stress factor [33]. We evaluated hydrogen peroxide (H_2_O_2_) and proline content in the leaves of both *Gh_A07G0747 (TOM1)* gene overexpressed lines and wild types under drought and cold stress conditions. Proline is a well-known osmoprotectant and antagonistic to H_2_O_2_ [34]. The H_2_O_2_ concentration was significantly higher in the leave of the wild type, and, prospectively, the proline levels were significantly lower in the leaves of the *Gh_A07G0747 (TOM1)* gene overexpressed lines (Fig. 2a). The proline concentration level showed no significant difference between the transgenic and the wild type under controlled condition but increased rapidly under drought and cold stress conditions in *Gh_A07G0747 (TOM1)* gene overexpressed line compared to the wild type. These results strongly confirmed that drought and cold stressed transgenic plants actively mounted a stress response when exposed to both drought and cold (at 4 °C) conditions. Moreover, the activities of glutathione (GSH) and ascorbate peroxidase (APX), exhibited continuously increasing trends, with approximately three-fold higher than the control, implying that these ROS-scavenging enzymes were responsible for the detoxification of ROS induced by drought and cold stress conditions in the transgenic lines. The two antioxidant enzymes were evaluated, glutathione (GSH) and ascorbate peroxidase (APX), and both showed significantly higher concentrations in the leaves of the transgenic lines (Fig. 2b-d). When plants are exposed to any form of stress, the production and accumulation of reactive oxygen undergo a drastic change, thus affecting the ability of the cell to convert the ROS into non-toxic levels [35]. The tolerant plants can induct more antioxidant enzymes to detoxify the ROS to non-toxic levels.

### Evaluation of germination rate, root elongation and biomass accumulation in transgenic and wild types under osmotic stress condition

**Fig. 3:**
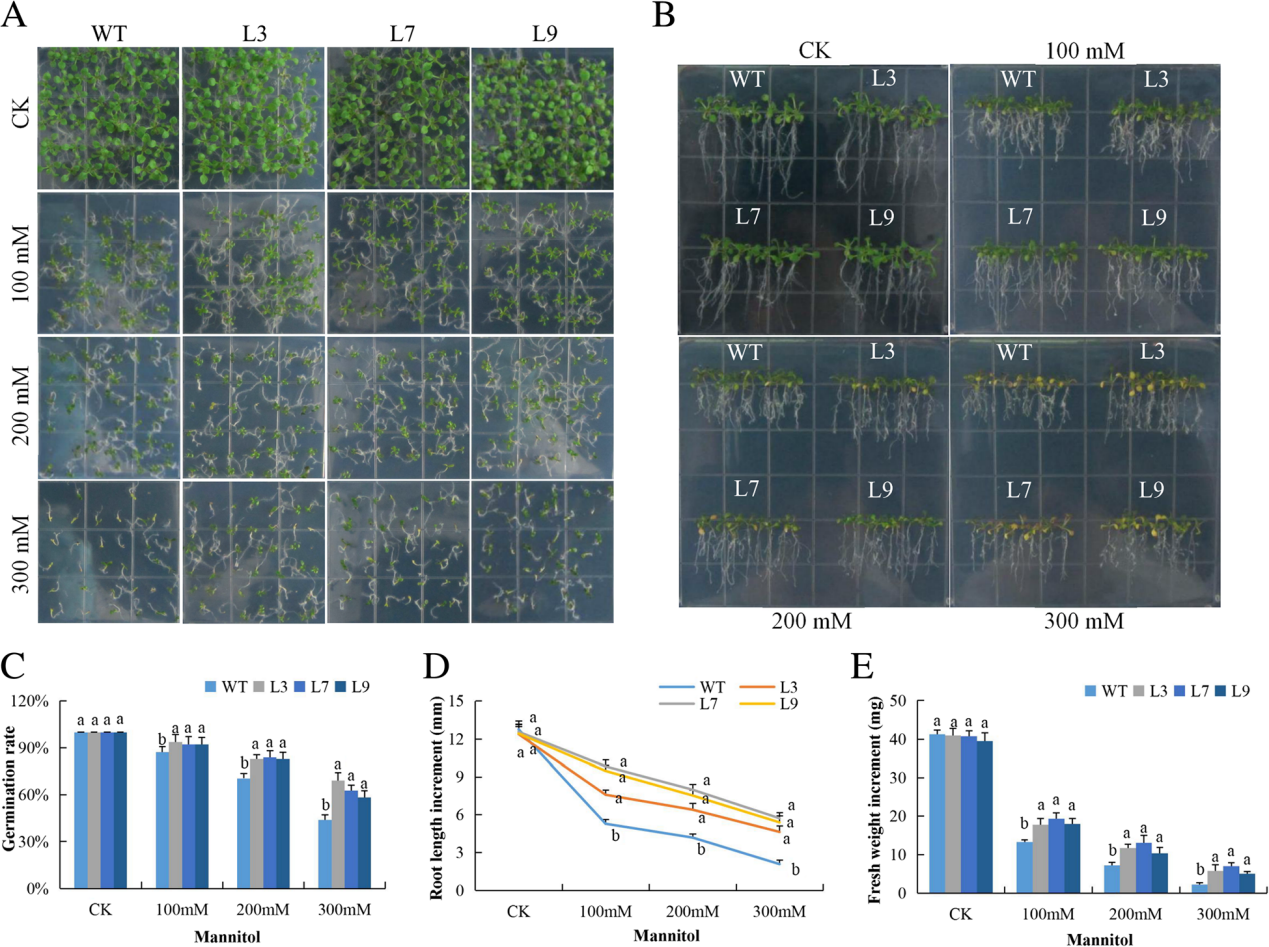
Overexpression of *Gh_A07G0747 (GhTOM1)* effects on germination, root growth and fresh biomass accumulation in transgenic plants and wild type under osmotic stress: **a** Seed germination in 0.5 MS supplemented with 0, 100, 200 and 300 mM mannitol for 10 days. **b** Root length evaluation in 0.5 MS medium supplemented with 0, 100, 200 and 300 mM mannitol. **c** Quantitative comparisons of germination rate of the transgenic and WT on mannitol 0.5 MS with (0, 100, 200 and 300 mM) for 10 days. **d** Root elongation comparisons on mannitol 0.5 MS with (0, 100, 200 and 300 mM). **e** Fresh weight determination. Each experiment was repeated three times. Bar indicates standard error (SE). The seedlings were scored and photographed after 6 days post germination. Different letters indicate significant differences between wild-type and OE lines (ANOVA; *p* < 0.05). CK: normal conditions

The germination rate of the seeds of the transgenic lines and the wild types showed significant variations under mannitol. Mannitol causes osmotic stress and thus results in artificial drought conditions. The rate of seed germination among the wild types compared to their controlled condition was approximately 20% reduction, and the percentage reduction increased to almost 70% at elevated concentration levels of mannitol (Fig. 3a). In the evaluation of the root growth among the transgenic and the wild types, we measure the roots for the two lines under drought stress, and there was a significant difference between the transgenic lines and the wild type. The roots of the transgenic lines had a higher growth index, with elongated roots (Fig. 3b). The level of root growth decreased with an increase in the concentration of mannitol, but despite the reduction in root lengths, the variation among the transgenic lines were insignificant compared to the differences exhibited between the transgenic lines and the wild type plants. The biomass accumulation between the transgenic lines and the wild type exhibited significant differences; the transgenic lines recorded relatively higher fresh biomass index compared to the wild types under various levels of mannitol concentrations (Fig. 3c-e).

### Evaluation of germination rate, root elongation and biomass accumulation in transgenic and wild types under ABA treatment

**Fig. 4:**
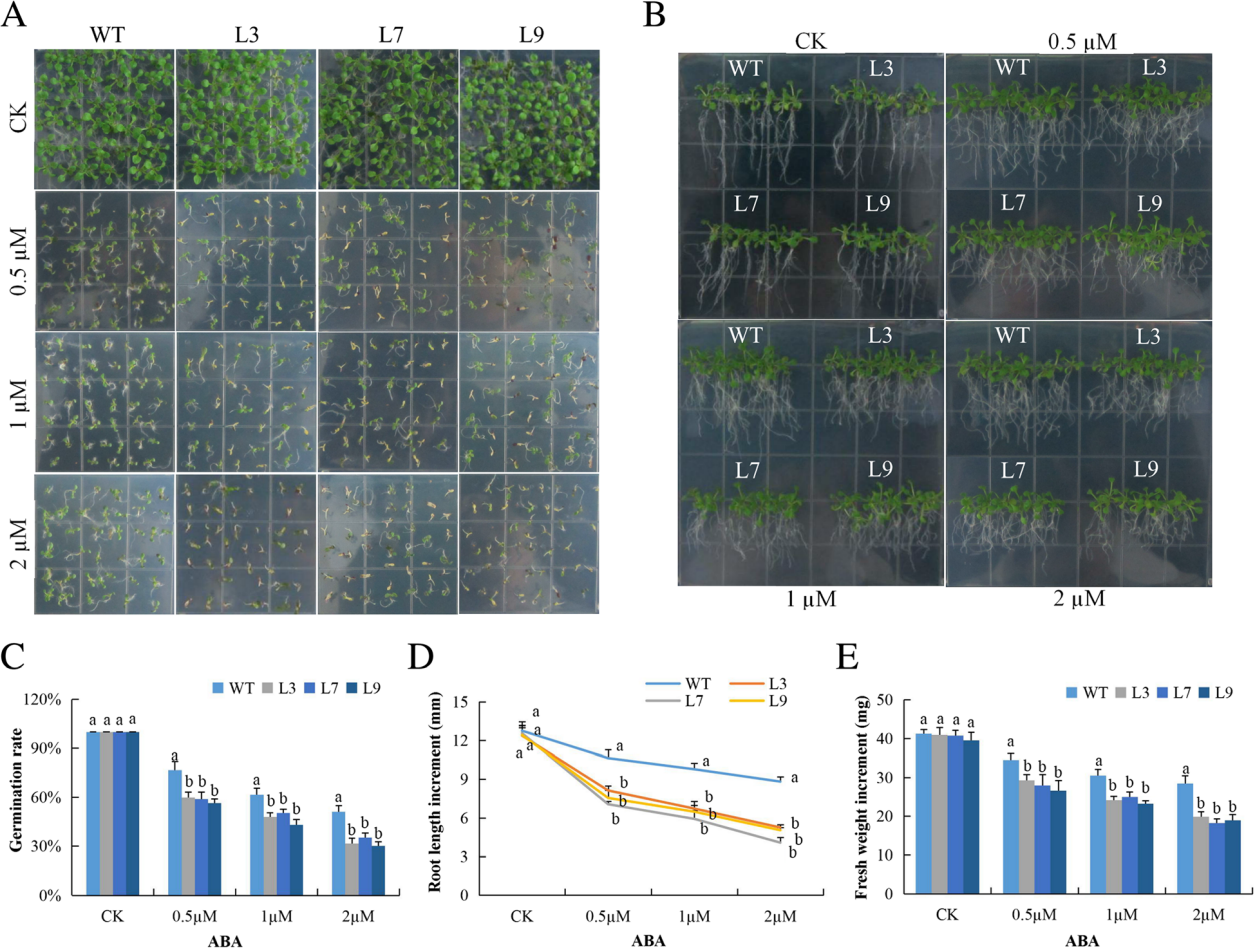
Overexpression of *Gh_A07G0747 (GhTOM1)* causes hypersensitivity to ABA-elicited seed germination, root growth inhibition and fresh biomass accumulation in transgenic plants and WT. **a** Seed germination rate of transgenic lines and wild type on ABA 0.5 MS with (0, 0.5, 1 and 2 μM) for 10 days. **b** Quantitative determination of root lengths. **c** Quantitative comparisons of germination rate of the transgenic and wild type on ABA 0.5 MS with (0, 0.5, 1 and 2 μM) after 10 days. **d** Root elongation comparisons on 0.5 MS with varied ABA concentrations (0, 0.5, 1 and 2 μM). **e** Quantitative determination of fresh weight biomass of wild-type and both overexpressed lines. The seedlings were scored and photographed after 6 days post germination. Each experiment was repeated three times. Bar indicates standard error (SE). Different letters indicate significant differences between wild-type and OE lines (ANOVA; *p* < 0.05). CK: normal conditions

ABA is involved in numerous responses in plant growth and development and an important plant phytohormone in stress responses [18]. The wild types were found to be more sensitive to ABA than the transgenic lines, being they exhibited lower germination rate under exogenous ABA condition (Fig. 4a), relatively lower root lengths (Fig. 4b) and decreased fresh biomass (Fig. 4c-e) than the transgenic lines. The results obtained here agreed with the previous reports, which showed that *GTG1* and *GTG2* overexpressed plants showed improved performance under stress compared to the wild types [17].

### Evaluation of germination rate, root elongation and biomass accumulation in transgenic and wild types under cold stress condition

**Fig. 5:**
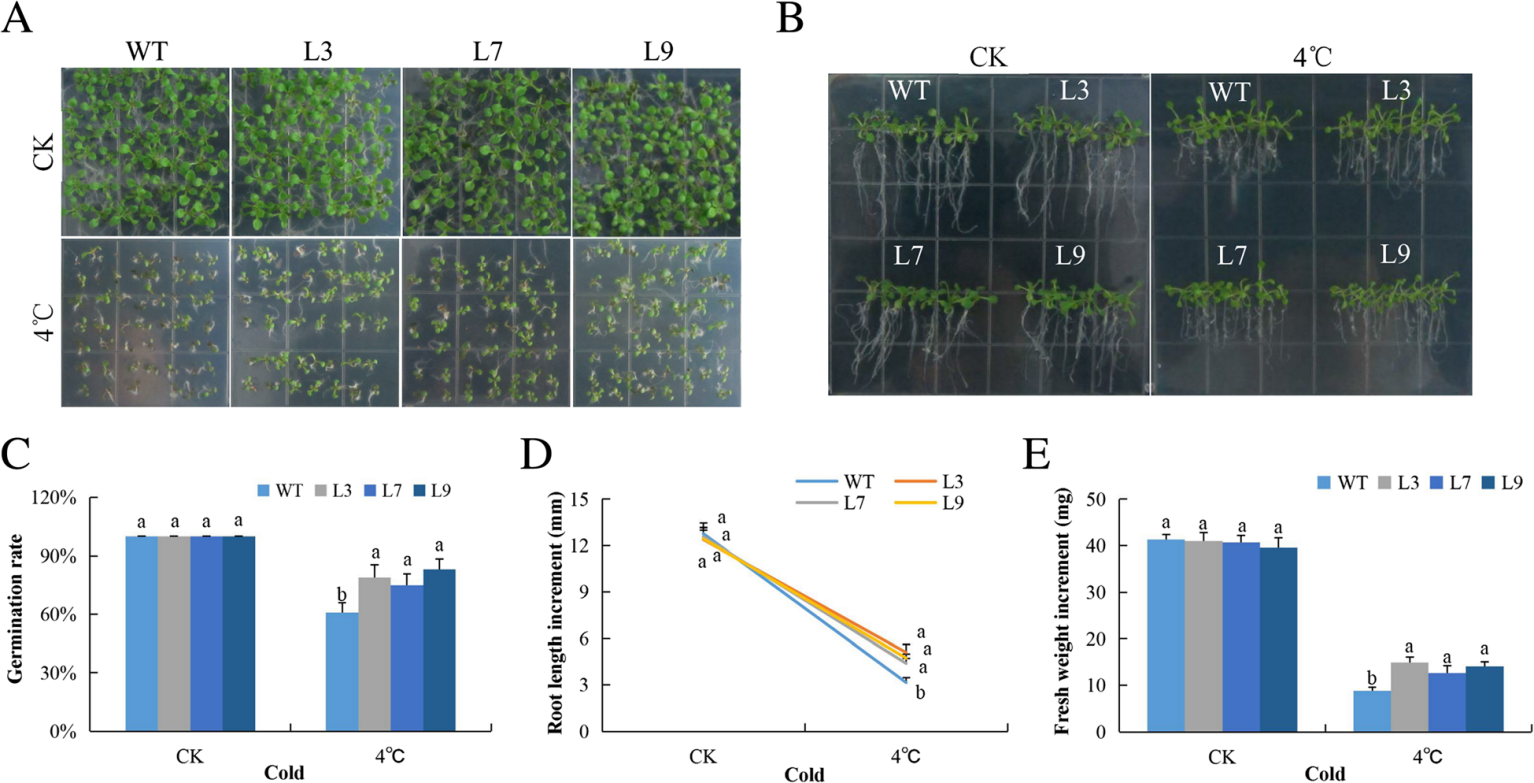
Overexpression of *Gh_A07G0747 (GhTOM1)* enhances cold stress tolerance: **a** Seed germination rate of transgenic lines and wild type on 0.5 MS at normal and low temperature (4 °C) for 10 days, in each set up, approximately 45 seeds were used to evaluate germination rate. **b** Quantitative determination of root lengths. **c** Quantitative comparisons of germination rate of the transgenic and wild type on 0.5MS at normal and low temperature (4 °C) for 10 days. **d** Root length evaluation on 0.5 MS at normal and low temperature (4 °C) for 10 days. **e** Quantitative determination of fresh weight biomass of wild-type and both overexpressed lines. The seedlings were scored and photographed after 6 days post germination. In each experiment three biological replications were done. Bar indicates standard error (SE). Different letters indicate significant differences between wild-type and OE lines (ANOVA; *p* < 0.05). CK: normal conditions

Germination rate was significantly higher among the seeds for transgenic plants (Fig. 5a-b); similarly, an increased root length and fresh biomass were observed among the transgenic lines compared to the wild types (Fig. 5c-e). Low temperature is a factor known to cause seed dormancy [36]. The increased germination rate of the seeds obtained from the transgenic lines under very low temperature (4 °C), showed that the gene could perhaps have a functional role, not only in breaking seed dormancy but also has a net effect on biomass accumulation under cold stress.

### The efficiency of gene silencing

After 12 day post inoculation (dpi) in seedlings of upland cotton (*G. hirsutum)* infected with Agrobacterium carrying the TRV-PDS construct, the albino phenotype was observed on the leaves and the stem region on the plants. Plants with the characteristic albino phenotype had a silencing efficiency of over 99% at 20 dpi due to the widespread of the chlorotic appearance on the leaf surface. To further determine the efficiency of VIGS, RT-qPCR assays were performed on leaves, stem and root tissues collected from the plants containing the TRV:00 empty vector and TRV:*Gh_A07G0747 (GhTOM1)-*construct. The transcript level of the target *Gh_A07G0747 (GhTOM1)* gene decreased in the *Gh_A07G0747 (GhTOM1)*-silenced plants compared with that in the TRV: 00 empty vector-infected plants in all tissues through VIGS were relatively higher on the leaves compared to other tissues (Additional file 6: Figure S4A-C). These results collectively suggested that the target gene was successfully knocked down in cotton plants.

### Expression of the silenced genes and evaluation of physiological and root traits in Gh_A07G0747 (TOM1)*-*silenced plants under drought and cold stress conditions

**Fig. 6:**
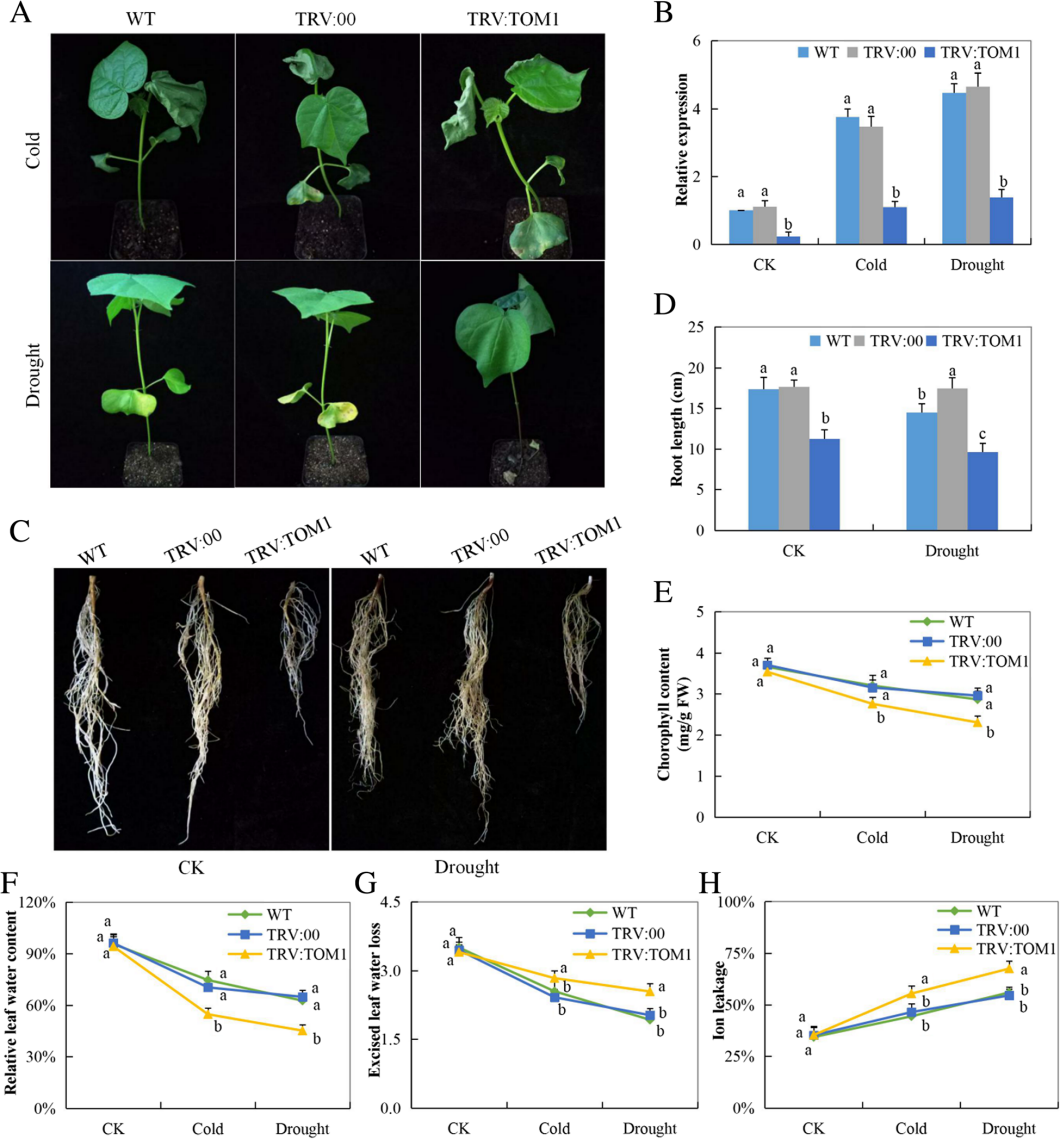
Evaluation of root and physiological parameters in the VIGS-treated cotton plants: **a** phenotypic evaluation of the mutant and wilt types under drought and cold stress conditions, photographs done after 8 days and 6 h under drought and cold stresses respectively, in each replication 12 plants were used. **b** RT-qPCR analysis of the change in the expression level of the *Gh_A07G0747 (GhTOM1)* gene on the leaf of the cotton plants treated with VIGS. **c** Root evaluation under drought stress, photographed after 8 days of stress exposure. **d** Root length determination. **e** Chlorophyll content in the TRV2 empty vector-carrying plants and *Gh_A07G0747 (GhTOM1)*-silenced resistant plants. **f** The excised leaf water loss (ELWL) level in the TRV2 empty vector-carrying plants and *Gh_A07G0747 (GhTOM1)*-silenced resistant plants. **g** The relative leaf water content (RLWC) in the TRV2 empty vector-carrying plants and *Gh_A07G0747 (GhTOM1)*-silenced resistant plants. **h** The cell membrane stability (CMS) evaluated through ion leakage in the TRV2 empty vector-carrying plants and *Gh_A07G0747 (GhTOM1)*-silenced resistant plants. Letter a/b indicated statistically significant differences (two-tailed, *p* < 0.05). Error bars of the represent the standard deviation of three biological replicates

The *Gh_A07G0747 (TOM1)* expression was significantly downregulated in the tissues of the VIGS plants compared to the wild type (Fig. 6a-b). Moreover, the root growth and fresh biomass evaluation of the plants under drought stress showed that the knockdown of the novel gene compromised the root growth and thus a significant reduction in both root length and biomass of the VIGS plants (Fig. 6c-d). The various traits measured were negatively affected in the *Gh_A07G0747 (GhTOM1)*-silenced plants compared to the wild and the positive control plants (Infused with empty vector) (Fig. 6e-h). The results obtained for the CMS, ELWL, Chlorophyll content and SLW showed that the VIGS plants were less tolerant of both drought and cold stresses compared to the controlled plants. The high ion leakage as observed on the VIGS plants under the stresses indicated that the plants had extensive cell damage leading to excessive ion leakage.

### Silencing of Gh_A07G0747 (GhTOM1)-gene compromised the ability of the cotton to tolerate drought and cold stress conditions

**Fig. 7:**
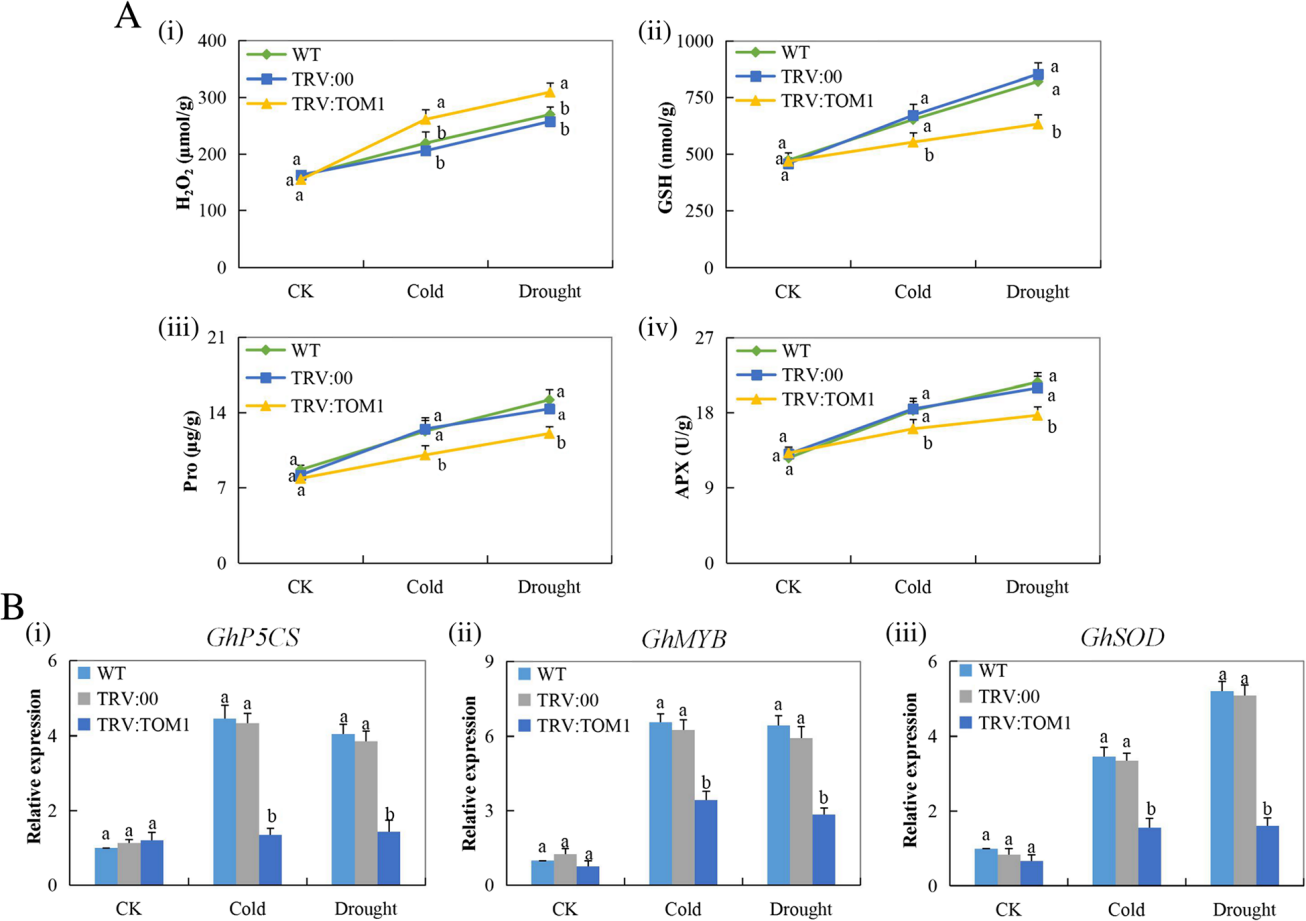
**a** The accumulation of oxidants and antioxidant enzymes in the VIGS-treated plants: (i-iv) The H_2_O_2_, GSH, Proline, APX content in the TRV2 empty vector-carrying plants and *Gh_A07G0747 (GhTOM1)*-silenced plants. **b**. RT-qPCR analysis of the change in the expression level of the *GhP5CS, GhMYB and GhSOD* stress resistant genes in cotton plants treated with VIGS. “TRV: 00” represents the plants carrying control the TRV2 empty vector, TRV: *Gh_A07G0747 (GhTOM1)* represents the *Gh_A07G0747 (GhTOM1)-*silenced plants. Letter a/b indicate statistically significant differences at *p* < 0.05. Error bars of the gene expression levels represent the standard deviation of three biological replicates

In upland cotton, evaluation of the oxidants and antioxidant enzymes showed that the *Gh_A07G0747 (GhTOM1)*-silenced plants were highly affected under drought and cold stresses compared to their wild types and the controlled plants. The antioxidants evaluated, proline, GSH and APX were significantly reduced in the leaves of *Gh_A07G0747 (GhTOM1)*-silenced plants under drought and cold stress conditions. Moreover, the concentration levels of oxidants were highly elevated in the *Gh_A07G0747 (GhTOM1)*-silenced plants compared with the wild types and controlled plants under the two stresses (Fig. 7a).

Evaluation of three known cotton stress-responsive genes, *GhMYB, GhSOD* and *GhP5CS*, on the *Gh_A07G0747 (GhTOM1)-*silenced plants, controlled and the wild types under drought and cold stress conditions. All the three genes exhibited a significant reduction in their expression levels in the leaves of the *Gh_A07G0747 (GhTOM1)*-silenced plants compared with the wild and the controlled plants (Fig. 7b). The downregulation of the three genes possibly indicated that the silenced gene had a functional effect in enhancing drought and cold stress tolerance.

## Discussion

Susceptible crops when exposed to water deficit and or extreme temperature condition, do results in negative effects on their growth and development, therefore limiting their production potentials in terms of yields and qualities [37]. Cotton being a world number one source of natural fiber, its production is threatened globally by climate change, the problem has been further compounded by the decreasing size of arable land for agricultural production as a result of increased demand for land for human settlement [38], and thus non-edible crops are being pushed to the regions with harsher climate and relatively low levels of precipitation [39, 40]. The known mechanism, adopted by the plants to improve their ability to tolerate arange of biotic and abiotic stress factors, is through the evolution of various plant transcription factors [41]. To date, various plant genes are playing an integral role in enhancing tolerance levels under drought and cold stresses, out of other forms of environmental stress factors. The survival of the plants under these extreme conditions requires efficient communication, and the ability of the plants to detect any slight changes within its environment [42]. One of the plant transcription factor known to play a role in signal detection, are the GPCR proteins [15]. The G-protein coupled receptors are known as the signal detectors not only in plants but also in animals [43]. In this study, we identified a novel gene, *Gh_A07G0747 (GhTOM1)* of GPCR type, transformed into the model plant and carried out functional analysis under drought and cold stress conditions. Moreover, we silenced the gene and reintroduce it to upland cotton, and evaluated the response of the mutant and non-mutant forms under drought and cold stress conditions.

The *Gh_A07G0747 (GhTOM1)* overexpressed plants showed higher germination rate under cold and osmotic stress conditions, but the germination rate was significantly reduced under ABA treatment compared to the wild types. A number of studies have shown that ABA is an important plant phytohormone, with diverse roles within the plant aimed at increasing their tolerance level to abiotic and biotic stress factors. Plants exposure to arranging of stresses has enabled them to acquire various mechanisms to surviving such stresses over a long evolutionary scale, one of which is the development of the ability to sensitively perceive incoming stresses and regulate their physiology accordingly [44]. Therefore, ABA plays a vital role in plant responses to both environmental and biotic stress factors, which occurs through rapid accumulation when plants are exposed to various stress factors and its rapid catabolism when plants are relieved of stress [45]. Moreover, ABA has been used extensively in tissue culture systems to promote somatic embryogenesis and improve somatic embryo quality by enhancing desiccation tolerance and preventing precocious germination [46]. The overexpression of the novel gene significantly affected the plant’s responses to ABA by exhibiting reduced root growth compared to the wild types, which showed that the plant *GPCR* genes are inducted through ABA independent signalling pathways, same has been observed for DREB plant transcription family with a functional role in enhancing stress tolerance in plants [47]. Similarly, NAC-type transcription factor SlNAC4 has been found to enhance salt and drought stress tolerance in tomato through an ABA-independent signaling network [48].

Plant acclimation to changes in their biospheres requires well-coordinated cellular homeostasis balance in all the various pathways in different plant cellular compartments [49]. Being the pathways are interdependent, any alteration in one affects others too, thus this delicate equilibrium does shift when plants are exposed to drought and cold stresses, more so when the cell or the plant is exposed to low water potential condition [50]. the changes in plant results in ROS imbalance, occasioned by excessive production of ROS [51]. ROS signals originating at different production sites within the cell have been shown to trigger large plant transcriptional changes and cellular reprogramming, which either protects the cell or does result in programmed cell death [52]. The ROS produced by the plants is unnecessary evil, which can be related to evapotranspiration process in plants, ROS serves as a signalling molecule which coordinates the stress responses, as well as growth and development of the plant [53]. Here in our study, overexpression of the novel *GPCR* gene enhanced a balanced response to excessive ROS due to the induction of more antioxidant enzymes, such as the GSH and APX, in addition to the high concentration of proline content. A number of studies have shown that, overexpression of stress-responsive genes, do results into high accumulation of antioxidants, for instance, overexpression of cotton *LEA2* genes resulted into two-folds in concentration of the antioxidant enzymes in the leaves of the transgenic lines compared to the wild type under drought stress condition [54].

The stable cell membrane under drought, and cold stresses depicts the ability of plant tissues to prevent electrolytes leakage by keeping the cell membrane in safe mood [55]. The evaluation of CMS, ELWL and SLW in this study showed that the transgenic plants showed higher adaptability to drought and cold stress conditions compared with their wild types under similar conditions. When plants are exposed to any form of abiotic stress, more drought and cold stress, inter and intracellular movement channels becomes disrupted thereby affecting other processes such as photosynthesis and respiration, thus decreasing the ability of plasma-lemma to retain the solute, which may be due to loosening of plasma membranes and increasing porosity [56]. Water deficit affects processes such as photosynthesis and photorespiration [57]. Some investigations have also shown that drought stress cause massive enzyme destruction related to various membranes, which are integral in maintaining the chemical balance in the cell under heat stress [58]. The ability of the transgenic lines to have relatively stable cell membrane as indicated by low level of electrolyte leakage showed that the gene had a functional role in maintaining the various membrane proteins. Furthermore, the GRAVY value of the protein encoded by the gene was less than 1 (0.35), which showed that the protein encoded by the plant *GPCR* gene of the *GhTOM1* domain was hydrophobic in nature. Hydrophobicity is highly associated to various plants stress-responsive genes, for instance over 99% of the proteins encoding the *LEA* genes in cotton have been found to hydrophobic in nature [54]. Hydrophobic proteins have protective functions to various cellular membranes against damage caused by oxidative stress effects. The proteins encoding the plasma membrane proteolipid 3 (*PMP3*) genes, are a class of small hydrophobic membrane proteins, known to be highly conserved among species [59]. The *PMP3* genes have been found to be highly upregulated in response to various stresses including drought, salinity and or cold, and participate in plant tolerance to abiotic stresses [60].

Analysis of the various stress-responsive genes in VIGS cotton plants and the gene overexpressed Arabidopsis plants, showed that the novel cotton *TOM1* gene of the GPCR domain, could be having a functional role in enhancing drought and cold stress tolerance in plants. In our previous research, we analysed the expression levels of four stress-responsive genes, such as *CBL1*, *ABF4*, *RD29A*, and *SOS2* genes, in which we found that, all the genes were highly upregulated in the leaf tissues of the overexpressed plants compared to their wild types [16]. Similarly, we analyzed some known plant stress-responsive genes such as *GhMYB, GhP5CS* and *GhSOD* genes, all the genes were downregulated in the VIGS cotton plants but were upregulated in the wild and controlled plants under drought and cold stress conditions. The downregulation of the three cotton stress-responsive genes clearly explained the rise in concentration of the oxidants and a decrease in antioxidant enzymes concentration in the leaves of the VIGS cotton plants under stress conditions.

## Conclusions

The findings of this research work have revealed that the plant GPCR protein plays a significant role in enhancing drought and cold stress tolerance. The transgenic lines exhibited higher CMS as evident by the low level of ion leakage under drought and cold stress conditions. Similarly, there was a low level of water loss on the excised leaves of the transgenic lines, with a high level of leaf saturation, as evaluated through SLW. When plants are exposed to any abiotic stress condition, the ROS production and its delicate balance within the cell is altered, increased ROS levels are lethal to the cell and can lead to cell death. The tolerant plants have been found to have the ability to induct more of the antioxidant enzymes to catalyze the ROS to non-toxic compounds, in the *Gh_A07G0747 (TOM1)* gene overexpressed lines, higher concentrations of the antioxidant enzymes were detected as compared to the wild type, further indicated that the gene had a functional role in enhancing drought and cold stress tolerance.

## Methods

### Identification and physiochemical property analysis of the TOM genes of the GPCR domain

Identification of the proteins encoded by the *TOM* genes of the GPCR domain was done by retrieving the PF06454 from the Pfam database. By use of the Pfam number, the TOM proteins for the three representative cotton species, *Gossypium hirsutum*, a tetraploid cotton genome was downloaded from Cotton Research Institute of Nanjing Agricultural University of China website (http://mascotton.njau.edu.cn), *Gossypium raimondii* genome from Phytozome 12 (http://www.phytozome.net/) and *Gossypium arboreum* was obtained from the Beijing Genome Institute of China (https://www.bgi.com/), with E-value < 0.01. The TOM domains were further confirmed by use of two online tools, SMART (http://smart.embl-heidelberg.de/) and ScanProsite tool (http://prosite.expasy.org/scanprosite/). Finally the physiochemical properties and subcellular localization prediction of the proteins encoded by the *TOM* genes were estimated by use of an online ExPASy Server tool (http://www.web.expasy.org/compute_pi/) and Wolfpsort (https://www.wolfpsort.hgc.jp/).

### Phylogenetic analysis

The three cotton GPCRs protein sequences together with the protein sequences for *A. thaliana*, *Oryza sativa* and *Theobroma cacao* were used in investigating the evolutionary history by using the neighbor-joining (NJ) method [61]. The bootstrap consensus tree was done at 1000 replicates to represent the evolutionary history of the taxa analyzed trough MEGA6 (http://www.megasoftware.net/mega.html). The multiple sequence alignment was performed by the Muscle module a component of MEGA6, adopting the default parameters: with open gap = − 2.9; gap extension penalty = 1 and hydrophobicity multiplier = 1.2.

### GO analysis, miRNA prediction and *Cis-*regulatory element analysis of the cotton GPCR proteins

All the protein sequences for the upland cotton, GPCR proteins were analysed. The proteins sequences were analysed through an online tool Blast2GO (https://www.blast2go.com), in the analysis of the late embryogenic abundant (LEA) proteins in cotton. The *GPCR* genes targeted by miRNAs were predicted by searching 5′ and 3′ UTRs and the CDS of all the genes for complementary sequences of the cotton miRNAs using the psRNATarget server with default parameters (http://plantgrn.noble.org /psRNATarget/?function = 3). The *Cis-*regulatory element sequences 1 kb up/down translation sites of all the upland cotton *GPCR* genes were predicted by an online tool, the PLACE database (http://www.dna.affrc.go.jp/PLACE/signals can.html).

### Plant materials and treatment

Two plants, cotton and Arabidopsis, were used in this research work. ICR-12, a major upland cotton (*G. hirsutum*) cultivar grown in most areas in China, was developed by Institute of Cotton Research, Chinese Academy of Agricultural Sciences (ICR-CAAS, thus the coding ICR). The model plant, *A. thaliana* ecotype Colombia-0 (Col-0) was used for the transformation of the novel cotton *GPCR* gene. The transformed T3 homozygous *Gh_A07G0747 (TOM1)* gene overexpressed lines and their wild types seeds were. The seeds were then plated on 0.5 MS medium. Plants were stratified at 4 °C in a dark chamber for a period of 3 days and then moved to well-conditioned growth chamber at 22 °C with a 16 h light/8 h dark photoperiod. After 7 days, the seedlings were transplanted in soil containing equal proportions of vermiculite, in 8 cm pots. Drought and cold stresses were imposed after 21 days of growth. Water was withdrawn for drought treated plants and for cold, the pots were shifted to cold chamber at 4 °C.The chlorophyll content evaluation was done after eight (8) days, while phenotypic traits evaluation was carried out after 16 days post stress treatment.

### Real-time quantitative PCR and standard RT-qPCR

Standard 20-mL PCR reactions were performed using BioMix Red (Bioline), forward (F) and reverse (R) primers with 1 mL of first-strand synthesis cDNA. the RT-qPCR conditions were programmed as described by Stephenson and Terry [62]. SYBR Green (Finnzymes) was used to monitor cDNA amplification with an Opticon DNA Engine Continuous Fluorescence Detector (GRI Ltd.). Specific primer, *Gh_A07G0747 (GhTOM1)* forward 5’TGCGAAAGCTTTTTCATCATTGG3’ and *Gh_A07G0747 (GhTOM1)* reverse 5’ACTTGTAGACGGGGCTGGTA3’ were designed and used to quantify expression of the overexpressed gene. The RT-qPCR was performed at 95 °C for 10 min followed by 35 cycles of 95 °C for 15 s and 60 °C for 1 min. All data were standardized by normalizing to *AtActin2* gene (F: 5’TTGTGCTGGATTCTGGTGATGG3’ and the R: 5’CCGCTCTGCTGTTGTG GTG3’ expression and analyzed using Opticon software.

### Germination assay under cold, ABA and Mannitol treatment

Germination of the transgenic and wild type seeds were evaluated under cold, ABA and osmotic stress initiated by mannitol application. WT Arabidopsis seeds and *Gh_A07G0747 (TOM1)* gene overexpressed line seeds were surface sterilized and placed on 0.5 MS media supplemented with mannitol (100 mM, 200 mM and 300 mM) and ABA (0.5 μM, 1 μM and 2 μM). The results were obtained from 3 biological replicate with 50 seeds each collected from independent seeds stocks. The seeds were plated on 0.5 MS media containing, ABA and mannitol. Following plating, seeds were stratified in the dark at 4 °C for 3 days and then transferred into 16 h light/8 h dark cycles at 22 °C. For cold stress, the seeds remained under low temperature of 4 °C, for a period of 10 days. Germination was determined by monitoring the emergence of the radicle at 10 days from 50 seeds per genotype per plate with three technical replicates for each of three biological repeats.

### Root assay under drought, cold and ABA treatments

Sterilized seeds were plated on 0.5 MS and put in a well-conditioned chamber, set at 4 °C for 3 days, and then placed vertically into 16 h light/8 h day cycles at 22 °C for 6 days. Seedlings were then transferred to new 0.5 MS media containing 0, 0.5, 1, and 2 μM ABA and grown vertically for a further 6 days. For cold stress condition, the seedlings were transferred to low temperature of 4 °C, for 6 days. Finally, under drought stress condition, Seedlings were then transferred to new 0.5 MS media containing 0, 100, 200, and 300 mM of mannitol, and grown vertically for a further 6 days. For analysis, photographic images of plates were taken and seedling root lengths measured using ImageJ software (http://rsbweb.nih.gov/ij/).

### Cloning of Gh_A07G0747 (GhTOM1), a GPCR novel gene and Production of expression constructs

The development of the T3 generation, with the expressed novel *GPCR* gene as done as previous work described in our previous research work, on the elucidation of the role of *GPCR* in plants under salt stress [16]. The transformation and primers requirements as described by Lu et al. [16].

### Virus induced gene silencing of the Gh_A07G0747 (GhTOM1) gene in upland cotton plant, *G. hirsutum*

For VIGS silencing of *Gh_A07G0747 (GhTOM1)*, the tobacco rattle virus (TRV)-based VIGS system was employed as described by Magwanga et al. [39] in evaluating the knockdown of two novel *CYP450* genes in cotton under drought and salt stress conditions. A 375 bp gene-specific fragment from *Gh_A07G0747* was amplified by PCR using *Gh_A07G0747 (GhTOM1)* 5′CGAGCTCTTCGTTATTGTGGTCATTGTG3′, *Sac1* restriction site and *Gh_A07G0747 (GhTOM1)* 5′CCTCGAGATTACTTGTAGACGGGGCTGG3′, *Xho1* restriction site primers. The PCR product was cloned into the pTRV2 vector to produce TRV: *Gh_A07G0747 (GhTOM1)*. Then the recombinant plasmids pTRV1, pTRV2, and TRV: *Gh_A07G0747 (GhTOM1)* were later transformed in *Agrobacterium tumefaciens* strain LBA4404 [63]. VIGS-infiltrated seedlings were allowed to grow for 2 weeks until the two-true-leaf stage and then leaves were collected and stored at − 80 °C. Seedlings were then simultaneously subjected to drought and cold stress treatments. For drought stress condition, irrigation was totally withdrawn, then after eight (8) days, various morphological, morphological and biochemical parameters were determined. And for cold stress, the seedlings were transferred into a cold chamber at 4 °C, for 6 h, after which evaluation was carried out. The root length was determined after eight (8) days of drought stress exposure.

We further analysed the expression levels of three stress-responsive genes, *GhMYB* forward sequence 5’TGGGAGTAGAGGAGGAGAAGC3’ and reverse sequence 5’TTGAGGTGCCTGTGGATTG3’; *GhP5CS* forward “TTGAAATAGTGGACGACGTGGC” and reverse sequence 5’CTCAGCGCCTAGACCAAAT CG3’ and the lastly *GhSOD* with forward 5’CATCTCTCACGCACTCTGTC3’ and reverse sequence 5’CCTTAGCCAT TTCTGTCTGTG3’. *GhActin* gene forward sequence 5’ATCCTCCGTCTTGACCTTG3’ and reverse sequence 5’TGTCCGTCAGGCAACT CAT3’ was used as the reference standardization for the RT-qPCR analysis. Finally, we analyzed the leaves of the wild plants, TRV:00 and *TRV:Gh_A07G0747(GhTOM1)* silenced plants, by use of their gene specific primers; TRV:*Gh_A07G0747 (GhTOM1)* forward sequence 5’GAAGATTAATGGCATAAAA3’ and the reverse sequence 5’ATAGTCCAAAAGCGAAGGG3’; the TRV1 forward sequence 5’TTACAGGTTATTTG GGCTAG3’ and the reverse sequence 5’CCGGGTTCAATTCCTTATC3’, and TRV2 forward 5’TGTTTGAG GGAAAAGTAGAGAACGT3’ and reverse 5’TTACCGATCAATCAAGATCAGTCGA3’ analysed through gel electrophoresis, the PCR conditions were programed as described by Winter et al., [64].

### Evaluation of oxidants, antioxidants, morphological and physiological parameters of the transgenic and wild types under drought and cold stress conditions

In order to understand the response mechanism of the transgenic and wild type plants to drought and cold stress conditions. We evaluated the various levels of oxidant and antioxidant enzymes, such as hydrogen peroxide (H_2_O_2_), proline, glutathione (GSH) and ascorbate peroxidase (APX). In addition to the various oxidants and antioxidants enzymes quantifications, we evaluated the nature of cell membrane stability (CMS), excised leaf water loss (ELWL) and saturated leaf weight (SLW). In all the various variables, were determined as described in the analysis of various physiological traits in *LEA2* gene overexpressed Arabidopsis plant [54], in the evaluation of the role of *LEA2* genes in enhancing root length under drought stress condition.

## Additional files

Additional file 1 : **Table S1** Identification and physiochemical properties of the proteins encoded by the TOM genes of the GPCR domain in the three cotton species, *G. hirsutum* (AD), *G. arboreum* (AA) and *G. raimondii* (DD). (DOCX 27 kb)

Additional file 2 : **Table S2** Gene ontology analysis of the *TOM* genes of the GPCR domain in cotton genomes. (DOCX 18 kb)

Additional file 3 : **Figure S1** Phylogenetic tree analysis of the various GPCR proteins identified in cotton and other plants. Thecc: *Theobroma cacao*, LOC: *Oryza sativa*, AT: *Arabidopsis thaliana*; Gorai: *Gossypium raimondii*; Gh: *Gossypium hirsutum* and Ga: *Gossypium arboreum (TIF 1468 kb)*

Additional file 4 : **Figure S2** *Cis-*regulatory element analysis and Gene Ontology analysis of the upland cotton *GPCR* gene. (A): The various *Cis-*regulatory elements detected for *Gh_A07G0747* overexpressed gene. (B): Gene ontological analysis (TIF 1098 kb)

Additional file 5 : **Figure S3** RNA seq. expression and RT-qPCR analysis of the expression of the cloned gene: (**A**) RNA sequence profiling under drought stress condition. **(B)** RNA sequence expression profiling under cold stress condition. **(C)** RT-qPCR validation of the cloned gene in various cotton tissues under normal condition. (**D**) RT-qPCR analysis performed to check 978 bp coding sequence integration in transformed T_0_ generation, number 1–13 transgenic lines, 14 positive control (*pWM101-Gh_A07G0747* (*GhTOM*)), and 15 is the negative control (wild-type, WT). **(E)** RT-qPCR analysis of the transgenic lines. The heat map was visualized using Mev.exe program (Showed by log 2 values) in control and in treated 1 h, 3 h, 6 h and 12 h after drought (PEG) and cold stresses. (i) Yellow–upregulated, blue–downregulated and black-no expression. (TIF 1301 kb)

Additional file 6 ; **Figure S4** Phenotype observed in the silenced plants with the TRV: 00 empty vector, wild type plants and *Gh_A07G0747 (GhTOM1)*-silenced plants at 12 days post inoculation: (A). No obvious symptoms in the leaves of the TRV: 00-infected plant (B). RT-qPCR analysis of the change in the expression level of the *Gh_A07G0747 (GhTOM1)* gene in cotton plants treated with VIGS. “TRV: 00” represents the plants carrying control the TRV2 empty vector; “TRV: *Gh_A07G0747 (GhTOM1)*” represents the *Gh_A07G0747 (GhTOM1)-*silenced plants. Letters a/b indicate statistically significant differences (two-tailed, *p* < 0.01). Error bars of the *Gh_A07G0747 (GhTOM1)* gene expression level represent the standard deviation of three biological replicates. (TIF 615 kb)

## Data Availability

All the relevant data and supplementary data are all availed including the primer sequences used in carrying out the RT-qPCR validation of the significant genes mined for the vital QTLs detected in this research work. All supplementary data supporting this research work are all made available in a public data repository and can be accessed

## Abbreviations

APX: Ascorbate peroxidase
CHLH: Chelatase H subunit
CMS: Cell membrane stability
ELWL: Excised leaf water loss
FCA: Flowering time control protein A
GDP: Guanosine diphosphate
GO: Gene ontology
GPCR: G-protein coupled receptor
GSH: Glutathione
GTP: Guanosine triphosphate
H_2_O_2_: Hydrogen peroxide
HHP: Heptahelical protein
MLO: Mildew-resistance locus O
ROS: Reactive oxygen species
SLW: Saturated leaf weight
TOM: Tobamovirus replication protein
