# Supplementary material for: Genome-wide analysis of the cotton G-coupled receptor proteins (GPCR) and functional analysis of GTOM1, a novel cotton GPCR gene under drought and cold stress
Source: BMC Genomics. 2019 Aug 14;20:651. doi: 10.1186/s12864-019-5972-y (PMC6694541; doi:10.1186/s12864-019-5972-y)
Supplement: Supplementary file 1 — : Table S1 Identification and physiochemical properties of the proteins encoded by the TOM genes of the GPCR domain in the three cotton species, G. hirsutum (AD), G. arboreum (AA) and G. raimondii (DD). (DOCX 27 kb) [file 12864_2019_5972_MOESM1_ESM.docx]

Supplementary Table 1: Identification and physiochemical properties of the proteins encoded by the TOM genes of the GPCR domain in the three cotton species, G. hirsutum (AD), G. arboreum (AA) and G. raimondii (DD).

| **Genome** | **Gene ID** | **Gene Name** | **Description** | **Chro.** | **Start** | **End** | **Strand** | **Length (bp)** | **Protein Length (aa)** | **MW (kDa)** | **Charge** | **pI** | **GRAVY** |
| --- | --- | --- | --- | --- | --- | --- | --- | --- | --- | --- | --- | --- | --- |
| AD | Gh_A03G1529 | TOM1 | Tobamovirus multiplication protein 1 | A03 | 95,951,060 | 95,954,035 | - | 2,976 | 271 | 31.478 | 12.5 | 9.352 | 0.579 |
|  | Gh_A04G1253 | TOM3 | Tobamovirus multiplication protein 3 | A04 | 62,591,785 | 62,597,706 | - | 5,922 | 272 | 31.521 | 3.5 | 7.574 | 0.599 |
|  | Gh_A05G1440 | TOM1 | Tobamovirus multiplication protein 1 | A05 | 14,950,490 | 14,953,714 | + | 3,225 | 291 | 33.687 | 9 | 9.041 | 0.661 |
|  | Gh_A07G0747 | TOM1 | Tobamovirus multiplication protein 1 | A07 | 11,687,587 | 11,693,293 | + | 5,707 | 325 | 37.084 | 23 | 10.01 | 0.35 |
|  | Gh_A10G0365 | TOM1 | Tobamovirus multiplication protein 1 | A10 | 3,340,797 | 3,347,772 | - | 6,976 | 396 | 44.238 | 3 | 7.122 | 0.464 |
|  | Gh_A12G1438 | TOM3 | Tobamovirus multiplication protein 3 | A12 | 73,175,985 | 73,183,181 | - | 7,197 | 268 | 30.921 | 5.5 | 8.207 | 0.609 |
|  | Gh_A13G0241 | TOM3 | Tobamovirus multiplication protein 3 | A13 | 2,823,366 | 2,827,544 | + | 4,179 | 289 | 33.603 | 18 | 10.19 | 0.58 |
|  | Gh_A13G0596 | TOM1 | Tobamovirus multiplication protein 1 | A13 | 14,111,783 | 14,113,994 | - | 2,212 | 224 | 25.927 | 12.5 | 9.84 | 0.627 |
|  | Gh_D04G1878 | TOM3 | Tobamovirus multiplication protein 3 | D04 | 51,121,020 | 51,126,349 | - | 5,330 | 282 | 32.851 | 5 | 7.992 | 0.576 |
|  | Gh_D05G1613 | TOM1 | Tobamovirus multiplication protein 1 | D05 | 14,554,705 | 14,557,876 | + | 3,172 | 291 | 33.705 | 9 | 9.041 | 0.652 |
|  | Gh_D10G0373 | TOM1 | Tobamovirus multiplication protein 1 | D10 | 3,299,454 | 3,305,915 | - | 6,462 | 377 | 42.767 | 2 | 7.04 | 0.351 |
|  | Gh_D11G2418 | TOM3 | Tobamovirus multiplication protein 3 | D11 | 48,310,923 | 48,311,606 | + | 684 | 112 | 12.826 | 6 | 8.494 | 0.565 |
|  | Gh_D12G1556 | TOM3 | Tobamovirus multiplication protein 3 | D12 | 46,513,482 | 46,522,251 | - | 8,770 | 293 | 34.201 | 8 | 8.858 | 0.422 |
|  | Gh_D13G0257 | TOM3 | Tobamovirus multiplication protein 3 | D13 | 2,477,467 | 2,481,698 | + | 4,232 | 289 | 33.635 | 17 | 10.11 | 0.552 |
|  | Gh_D13G0530 | TOM1 | Tobamovirus multiplication protein 1 | D13 | 6,994,712 | 7,002,690 | + | 7,979 | 282 | 32.837 | 23.5 | 10.33 | 0.37 |
|  | Gh_Sca207201G | NA | NA | NA | NA | NA | NA | - | NA | NA | NA | NA | NA |
| AA | Ga03G2299 | TOM1 | Tobamovirus multiplication protein 1 | Chr03 | 131,182,379 | 131,185,357 | - | 2,979 | 271 | 31.453 | 11.5 | 9.333 | 0.594 |
|  | Ga05G1805 | TOM1 | Tobamovirus multiplication protein 1 | Chr05 | 16,471,741 | 16,474,980 | + | 3,240 | 291 | 33.705 | 9 | 9.041 | 0.652 |
|  | Ga07G0961 | Carnmt1 | Carnosine N-methyltransferase | Chr07 | 13,083,202 | 13,101,148 | + | 17,947 | 860 | 97.963 | 0.5 | 6.531 | -0.127 |
|  | Ga10G2668 | TOM1 | Tobamovirus multiplication protein 1 | Chr10 | 125,902,212 | 125,908,633 | + | 6,422 | 357 | 40.327 | 1 | 6.761 | 0.429 |
|  | Ga10G2950 | TOM1 | Tobamovirus multiplication protein 1 | Chr10 | 128,320,317 | 128,323,213 | + | 2,897 | 291 | 33.355 | 8.5 | 8.874 | 0.66 |
|  | Ga12G1285 | TOM3 | Tobamovirus multiplication protein 3 | Chr12 | 16,525,940 | 16,534,149 | + | 8,210 | 289 | 33.456 | 11.5 | 9.546 | 0.6 |
|  | Ga13G0287 | TOM3 | Tobamovirus multiplication protein 3 | Chr13 | 2,977,886 | 2,982,066 | + | 4,181 | 289 | 33.589 | 17 | 10.12 | 0.581 |
|  | Ga13G0552 | TOM1 | Tobamovirus multiplication protein 1 | Chr13 | 8,065,501 | 8,068,257 | + | 2,757 | 293 | 34.282 | 16.5 | 9.765 | 0.586 |
|  | Ga14G1735 | TOM3 | Tobamovirus multiplication protein 3 | tig00015727 | 382,895 | 387,643 | + | 4,749 | 290 | 33.8 | 8 | 8.816 | 0.611 |
| DD | Gorai.001G092500 | TOM1 | Tobamovirus multiplication protein 1 | Chr01 | 10,292,446 | 10,298,692 | + | 6,247 | 350 | 39.59 | 23 | 9.858 | 0.479 |
|  | Gorai.002G040200 | SAPK2 | Serine/threonine-protein kinase SAPK2 | Chr02 | 3,297,732 | 3,301,961 | + | 4,230 | 127 | 14.962 | 6 | 9.708 | 0.251 |
|  | Gorai.002G150900 | TOM3 | Tobamovirus multiplication protein 3 | Chr02 | 29,604,352 | 29,606,009 | + | 1,658 | 86 | 10.049 | 6 | 8.838 | 0.337 |
|  | Gorai.005G220700 | TOM1 | Tobamovirus multiplication protein 1 | Chr05 | 60,350,693 | 60,353,743 | - | 3,051 | 262 | 30.485 | 17 | 10.02 | 0.499 |
|  | Gorai.008G171700 | TOM3 | Tobamovirus multiplication protein 3 | Chr08 | 44,664,816 | 44,674,208 | - | 9,393 | 289 | 33.462 | 11.5 | 9.546 | 0.588 |
|  | Gorai.009G177100 | TOM1 | Tobamovirus multiplication protein 1 | Chr09 | 13,701,332 | 13,705,507 | + | 4,176 | 292 | 33.833 | 9 | 9.041 | 0.638 |
|  | Gorai.010G079300 | TOM3 | Tobamovirus multiplication protein 3 | Chr10 | 11,503,072 | 11,504,804 | - | 1,733 | 92 | 10.504 | 4.5 | 8.753 | 0.753 |
|  | Gorai.011G015600 | TOM1 | Tobamovirus multiplication protein 1 | Chr11 | 1,095,851 | 1,099,556 | - | 3,706 | 291 | 33.382 | 8.5 | 8.872 | 0.698 |
|  | Gorai.011G042100 | TOM1 | Tobamovirus multiplication protein 1 | Chr11 | 3,118,826 | 3,125,957 | - | 7,132 | 357 | 40.321 | 0 | 6.501 | 0.432 |
|  | Gorai.012G184200 | TOM3 | Tobamovirus multiplication protein 3 | Chr12 | 35,143,300 | 35,151,941 | - | 8,642 | 289 | 33.67 | 9 | 9.044 | 0.574 |
|  | Gorai.013G028100 | THH1 | Protein TOM THREE HOMOLOG 1 | Chr13 | 2,095,047 | 2,098,979 | + | 3,933 | 243 | 28.437 | 12.5 | 9.965 | 0.595 |
|  | Gorai.013G061000 | TOM1 | Tobamovirus multiplication protein 1 | Chr13 | 6,622,078 | 6,625,118 | + | 3,041 | 293 | 34.287 | 15 | 9.636 | 0.548 |
