# Supplementary material for: Genome-wide analysis of the cotton G-coupled receptor proteins (GPCR) and functional analysis of GTOM1, a novel cotton GPCR gene under drought and cold stress
Source: BMC Genomics. 2019 Aug 14;20:651. doi: 10.1186/s12864-019-5972-y (PMC6694541; doi:10.1186/s12864-019-5972-y)
Supplement: Supplementary file 2 — : Table S2 Gene ontology analysis of the TOM genes of the GPCR domain in cotton genomes. (DOCX 18 kb) [file 12864_2019_5972_MOESM2_ESM.docx]

Supplementary Table 2: Gene ontology analysis of the *TOM* genes of the GPCR domain in cotton genomes.

| **Genome type** | **Gene ID** | **Description** | **GO numbers** | **GO IDs** | **GO Names** |
| --- | --- | --- | --- | --- | --- |
| AA | Cotton_A_00801.1 | tobamovirus multiplication protein 1-like | 1 | C:GO:0016021 | C:integral component of membrane |
|  | Cotton_A_00877.1 | tobamovirus multiplication protein 3-like | 2 | C:GO:0016021; P:GO:0051301 | C:integral component of membrane; P:cell division |
|  | Cotton_A_04698.1 | tobamovirus multiplication protein 1-like | 1 | C:GO:0016021 | C:integral component of membrane |
|  | Cotton_A_11729.1 | tobamovirus multiplication protein 3-like | 2 | C:GO:0016021; P:GO:0051301 | C:integral component of membrane; P:cell division |
|  | Cotton_A_17563.1 | tobamovirus multiplication protein 1-like isoform X1 | 1 | C:GO:0016021 | C:integral component of membrane |
|  | Cotton_A_24028.1 | tobamovirus multiplication protein 1 isoform X1 | 1 | C:GO:0016021 | C:integral component of membrane |
|  | Cotton_A_31066.1 | protein TOM THREE HOMOLOG 1 | 2 | C:GO:0016021; P:GO:0051301 | C:integral component of membrane; P:cell division |
| DD | Gorai.001G092500.1 | tobamovirus multiplication protein 1-like isoform X1 | 1 | C:GO:0016021 | C:integral component of membrane |
|  | Gorai.002G150900.1 | tobamovirus multiplication protein 3-like isoform X2 | 1 | C:GO:0016021 | C:integral component of membrane |
|  | Gorai.005G220700.1 | tobamovirus multiplication protein 1-like isoform X1 | 1 | C:GO:0016021 | C:integral component of membrane |
|  | Gorai.008G171700.1 | protein TOM THREE HOMOLOG 1 | 2 | C:GO:0016021; P:GO:0051301 | C:integral component of membrane; P:cell division |
|  | Gorai.009G177100.1 | tobamovirus multiplication protein 1-like | 1 | C:GO:0016021 | C:integral component of membrane |
|  | Gorai.010G079300.1 | tobamovirus multiplication protein 3-like isoform X2 | 1 | C:GO:0016021 | C:integral component of membrane |
|  | Gorai.011G015600.1 | tobamovirus multiplication protein 1 | 1 | C:GO:0016021 | C:integral component of membrane |
|  | Gorai.011G042100.1 | tobamovirus multiplication protein 1-like isoform X1 | 1 | C:GO:0016021 | C:integral component of membrane |
|  | Gorai.012G184200.1 | tobamovirus multiplication protein 3-like | 2 | C:GO:0016021; P:GO:0051301 | C:integral component of membrane; P:cell division |
|  | Gorai.013G028100.1 | tobamovirus multiplication protein 3-like | 2 | C:GO:0016021; P:GO:0051301 | C:integral component of membrane; P:cell division |
|  | Gorai.013G061000.1 | tobamovirus multiplication protein 1-like | 1 | C:GO:0016021 | C:integral component of membrane |
| AD | Gh_A03G1529.1 | tobamovirus multiplication protein 1-like | 1 | C:GO:0016021 | C:integral component of membrane |
|  | Gh_A04G1253.1 | tobamovirus multiplication protein 3-like | 2 | C:GO:0016021; P:GO:0051301 | C:integral component of membrane; P:cell division |
|  | Gh_A05G1440.1 | tobamovirus multiplication protein 1-like | 1 | C:GO:0016021 | C:integral component of membrane |
|  | Gh_A10G0365.1 | tobamovirus multiplication protein 1-like isoform X1 | 1 | C:GO:0016021 | C:integral component of membrane |
|  | Gh_A12G1438.1 | protein TOM THREE HOMOLOG 1 | 2 | C:GO:0016021; P:GO:0051301 | C:integral component of membrane; P:cell division |
|  | Gh_A13G0241.1 | tobamovirus multiplication protein 3-like | 2 | C:GO:0016021; P:GO:0051301 | C:integral component of membrane; P:cell division |
|  | Gh_A13G0596.1 | tobamovirus multiplication protein 1-like | 1 | C:GO:0016021 | C:integral component of membrane |
|  | Gh_D04G1878.1 | tobamovirus multiplication protein 3-like | 2 | C:GO:0016021; P:GO:0051301 | C:integral component of membrane; P:cell division |
|  | Gh_D05G1613.1 | tobamovirus multiplication protein 1-like | 1 | C:GO:0016021 | C:integral component of membrane |
|  | Gh_D10G0373.1 | tobamovirus multiplication protein 1-like isoform X1 | 1 | C:GO:0016021 | C:integral component of membrane |
|  | Gh_D11G2418.1 | tobamovirus multiplication protein 3-like isoform X2 | 1 | C:GO:0016021 | C:integral component of membrane |
|  | Gh_D12G1556.1 | tobamovirus multiplication protein 3-like | 2 | C:GO:0016021; P:GO:0051301 | C:integral component of membrane; P:cell division |
|  | Gh_D13G0257.1 | protein TOM THREE HOMOLOG 1 | 2 | C:GO:0016021; P:GO:0051301 | C:integral component of membrane; P:cell division |
